# Cognitive and Tactile Factors Affecting Human Haptic Performance in Later Life

**DOI:** 10.1371/journal.pone.0030420

**Published:** 2012-01-23

**Authors:** Tobias Kalisch, Jan-Christoph Kattenstroth, Rebecca Kowalewski, Martin Tegenthoff, Hubert R. Dinse

**Affiliations:** 1 Department of Neurology, BG-Kliniken Bergmannsheil, Ruhr-University Bochum, Bochum, Germany; 2 Neural Plasticity Lab, Institute for Neuroinformatics, Ruhr-University Bochum, Bochum, Germany; McMaster University, Canada

## Abstract

**Background:**

Vision and haptics are the key modalities by which humans perceive objects and interact with their environment in a target-oriented manner. Both modalities share higher-order neural resources and the mechanisms required for object exploration. Compared to vision, the understanding of haptic information processing is still rudimentary. Although it is known that haptic performance, similar to many other skills, decreases in old age, the underlying mechanisms are not clear. It is yet to be determined to what extent this decrease is related to the age-related loss of tactile acuity or cognitive capacity.

**Methodology/Principal Findings:**

We investigated the haptic performance of 81 older adults by means of a cross-modal object recognition test. Additionally, we assessed the subjects' tactile acuity with an apparatus-based two-point discrimination paradigm, and their cognitive performance by means of the non-verbal Raven-Standard-Progressive matrices test. As expected, there was a significant age-related decline in performance on all 3 tests. With the exception of tactile acuity, this decline was found to be more distinct in female subjects. Correlation analyses revealed a strong relationship between haptic and cognitive performance for all subjects. Tactile performance, on the contrary, was only significantly correlated with male subjects' haptic performance.

**Conclusions:**

Haptic object recognition is a demanding task in old age, especially when it comes to the exploration of complex, unfamiliar objects. Our data support a disproportionately higher impact of cognition on haptic performance as compared to the impact of tactile acuity. Our findings are in agreement with studies reporting an increase in co-variation between individual sensory performance and general cognitive functioning in old age.

## Introduction

### Haptic perception

Haptic object recognition is perfectly performed, countless times every day, as healthy adults can identify common and usual objects within 2–3 seconds with almost 100% accuracy [1]. Haptic perception is a process mediated by cutaneous and kinesthetic afferent subsystems [2]. A large number of mechanoreceptors and thermoreceptors embedded in the skin as well as mechanoreceptors in muscles, tendons, and articulated joints provide the information necessary for the active exploration of objects and surface properties [3]. This manual exploration process is based on a number of so-called exploratory procedures, i.e., highly stereotypical hand-movements, which are associated with certain object properties [4], [1]. Subjects who must recognize an object first employ a fast general exploratory procedure that provides an overview of multiple dimensions of the object. This overview is then used to decide which more specific exploratory procedures should be applied next in order to identify the object. The succession of selection and application of exploratory procedures will be repeated until the object is recognized [5].

Although vision is the primary sensory modality used by humans to explore and identify objects in their environment, haptic perception often provides the same information about certain characteristics of an object [6], as both modalities are based on the extraction of basic features such as the spatial arrangement of contours [7]. Macro geometric features such as object orientation, shape, and size, are predominantly processed by the visual system, even during haptic object exploration [8]. Visual and haptic object exploration are similarly impaired by changes in object characteristics such as orientation [9], size [10], [11], and surface properties [12]. There is much converging evidence showing broad similarities between visual object recognition and haptic object recognition, which are a consequence of substantial overlaps in the higher-order neural resources required for both types of perception [7], [13]–[15]. As there is only a relatively small body of literature investigating age-related changes in haptics [16], [17], we aimed to investigate the influence of reduced tactile acuity and cognitive capacity on the haptic performance of older adults.

### Age-related factors contributing to the loss of tactile acuity and cognition

Physiological brain aging is characterized by a number of alterations that provoke age-dependent decline of sensory processing, motor performance, and cognitive function [18]–[20].

Age-related changes develop at all stages of the somatosensory processing pathway. Skin conformance is altered [21], [22] and the density of Meissner's and Pacinian corpuscles decreases in old age [23]–[25], while Merkel-neurite complexes might possibly be less affected [23], [25]. Additionally, nerve conduction velocity and sensory nerve action potentials slow down [26]–[28]. These changes are thought to be due to an age-related reduction in the number and density of myelinated peripheral nerve fibers, as well as a decrease in thickness of the myelin in the remaining fibers [29], [30]. Furthermore, there is evidence of substantial decline in gray matter density of the aged human brain [31], [32]. Along with neuronal apoptosis and the loss of synaptic contacts described in some regions of the brain [33]–[35], as the brain ages, the concentrations of acetylcholine, noradrenaline, dopamine, and GABA and NMDA receptors [36] decrease. These changes, along with altered functional activation patterns [37], dramatically affect somatosensory information processing as has been repeatedly demonstrated for tactile discrimination performance [37]–[45]. Experiments in adults revealed that adding constraints to the manual exploration process, i.e. reducing cutaneous information (spatial, temporal and thermal) or kinesthetic information (spatial and temporal) significantly impairs haptic perception [46]. These experiments resemble to some extent conditions arising during the human aging process thereby highlighting the dramatic impact of age on haptic performance.

Cognitive aging manifests as a mild age-related decline in cognitive functions with highly individual changes in general cognitive capacity, as well as domain-specific declines in fluid reasoning, mental processing speed, episodic memory, and spatial ability [47]–[49]. The mechanisms that are thought to underlie these decreases fall into 2 general categories [19]. On the one hand, one global undifferentiated mechanism, such as cognitive processing-speed, could account for the loss of performance [50]. On the other hand, the age-related decline might be caused by specific cognitive mechanisms, such as executive functioning, which is used in service of many cognitive tasks, occurring in everyday life or work related tasks [51].

### Interdependence between sensorimotor functioning and cognition in later life

The majority of investigations into age-related decline of sensory, sensorimotor, and cognitive functions have looked at the components individually, but it is generally accepted that the loss of functional integrity between the domains is functionally coupled [52], [53]. In recent years, a number of studies have reported an increase in co-variation or interdependence between sensory and cognitive functions in old age [54], [55], [52], [56], [57]. Data from large-scale cross-sectional and longitudinal studies such as the Berlin aging study (BASE, [58]) showed strong relationships between intellectual and sensory functioning in old age [56]. Experimental studies investigating the relationship between sensory functions and cognition used either a simulated loss of sensation to explore the effects on cognitive function [59]–[61], or cognitive load manipulations on sensorimotor performance [62], [63]. In general, both interventions affected older adults' performance more than that of younger adults. Some authors hypothesized that sensory and sensorimotor declines may precede and predict cognitive decline [64], [65], whereas others refrained from assigning priority to any of the 3 domains, but favored either a common cause affecting all functions [52], an increase in cross-domain resource competition, or a combination of both [66].

Particularly in old age, sensory, sensorimotor, and cognitive performance determines the extent to which a mobile and independent life is possible [53]. For this reason, the investigation of the development of these processes into late adulthood is not only of general interest, but offers a direct link to gerontological practice [18], [19]. In the present study, we investigated the extent to which the age-related decline in haptic performance is related to the individual loss of tactile acuity and cognitive capacity.

## Results

### Haptic performance

**Figure 1:**
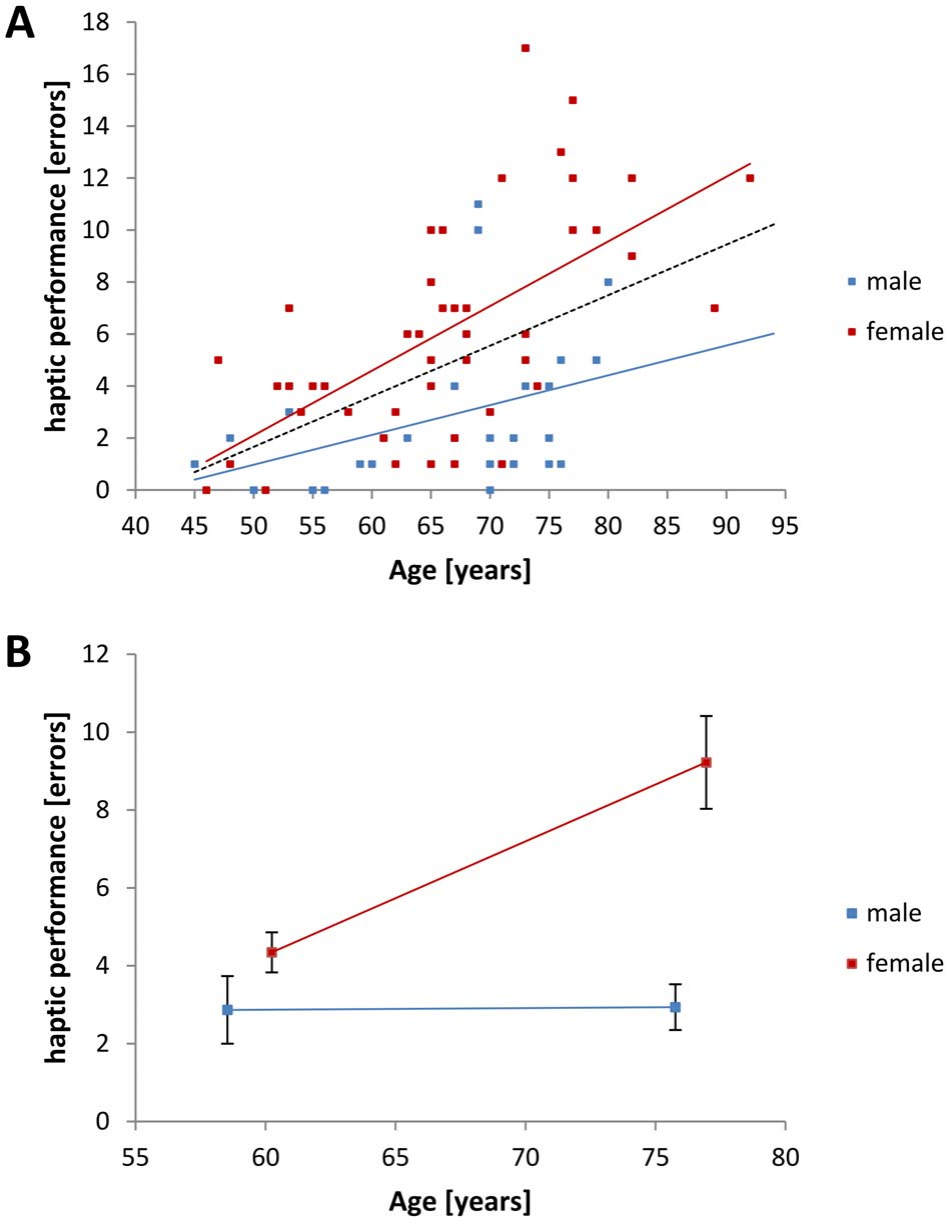
Development of haptic performance in later life. **A.** Individual haptic performance (i.e., number of errors) is depicted for male (blue squares, N = 31) and female (red squares, N = 47) subjects. Trend lines are inserted for male (solid blue), female (solid red), and all subjects (dashed black). Haptic performance declined with increasing age (Pearson correlation, N = 78, r = 0.479, p≤0.001). **B.** Group data for haptic performance (i.e., number of errors) for younger adults (left squares; females: N = 29, 60.24±7.07 years; males: N = 15, 58.53±7.62 years) and older adults (right squares; females: N = 20, 76.95±5.94 years; males: N = 17, 75.76±6.71 years). There was a significant interaction of the subjects' age and gender with their haptic performance (AGE*GENDER: F_(1,31)_ = 17.535; p≤0.001). Black bars indicate SEM.

The haptic performance of all subjects declined with increasing age (Pearson correlation, N = 78, r = 0.479, p≤0.001), with the decline being stronger in female subjects (Pearson correlation, N = 47, r = 0.585, p≤0.001) as compared to male subjects (Pearson correlation, N = 31, r = 0.417, p = 0.010) (**fig. 1A**). We found a significant interaction of the subjects' age and gender with their haptic performance (AGE*GENDER: F_(1,31)_ = 17.535; p≤0.001) indicating a stronger age-related increase of the number of errors in the cross-modal haptic task for female subjects (**fig. 1B**).

### Tactile performance

**Figure 2:**
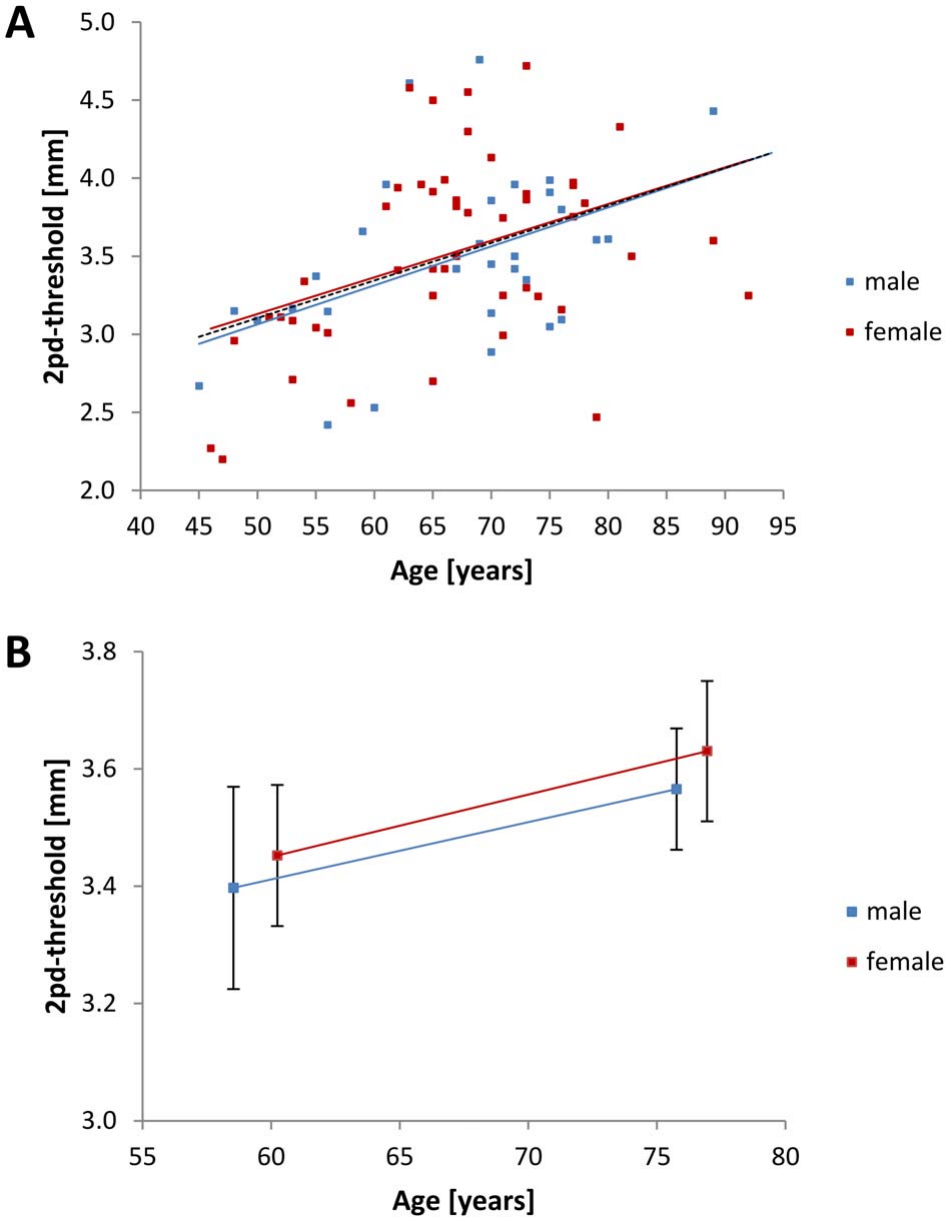
Development of tactile performance in later life. **A.** Individual two-point discrimination thresholds (i.e., inverse tactile acuity) are depicted for male (blue squares, N = 31) and female (red squares, N = 48) subjects. Trend lines are inserted for male (solid blue), female (solid red), and all subjects (dashed black). Tactile acuity declined with increasing age (Pearson correlation, N = 79, r = 0.430, p≤0.001). **B.** Group data for tactile performance (i.e., two-point discrimination threshold) for younger adults (left squares; females: N = 29, 60.24±7.07 years; males: N = 15, 58.53±7.62 years) and older adults (right squares; females: N = 20, 76.95±5.94 years; males: N = 17, 75.76±6.71 years). There was no significant interaction of the subjects' age and gender with their tactile acuity (AGE*GENDER: F_(1,31)_ = 17.535; p≤0.001). Black bars indicate SEM.

Tactile performance, as assessed by the two-point discrimination test, declined with increasing age (Pearson correlation, N = 79, r = 0.430, p≤0.001) in both male (Pearson correlation, N = 31, r = 0.469, p = 0.004) and female subjects (Pearson correlation, N = 48, r = 0.408, p = 0.002) (**fig. 2A**). For tactile performance, we found no interaction of the subjects' age and gender (AGE*GENDER: F_(1,32)_ = 2.942; p = 0.096) indicating almost the same degree of age-related decline in tactile performance for both male and female subjects (**fig. 2B**).

### Cognitive performance

**Figure 3:**
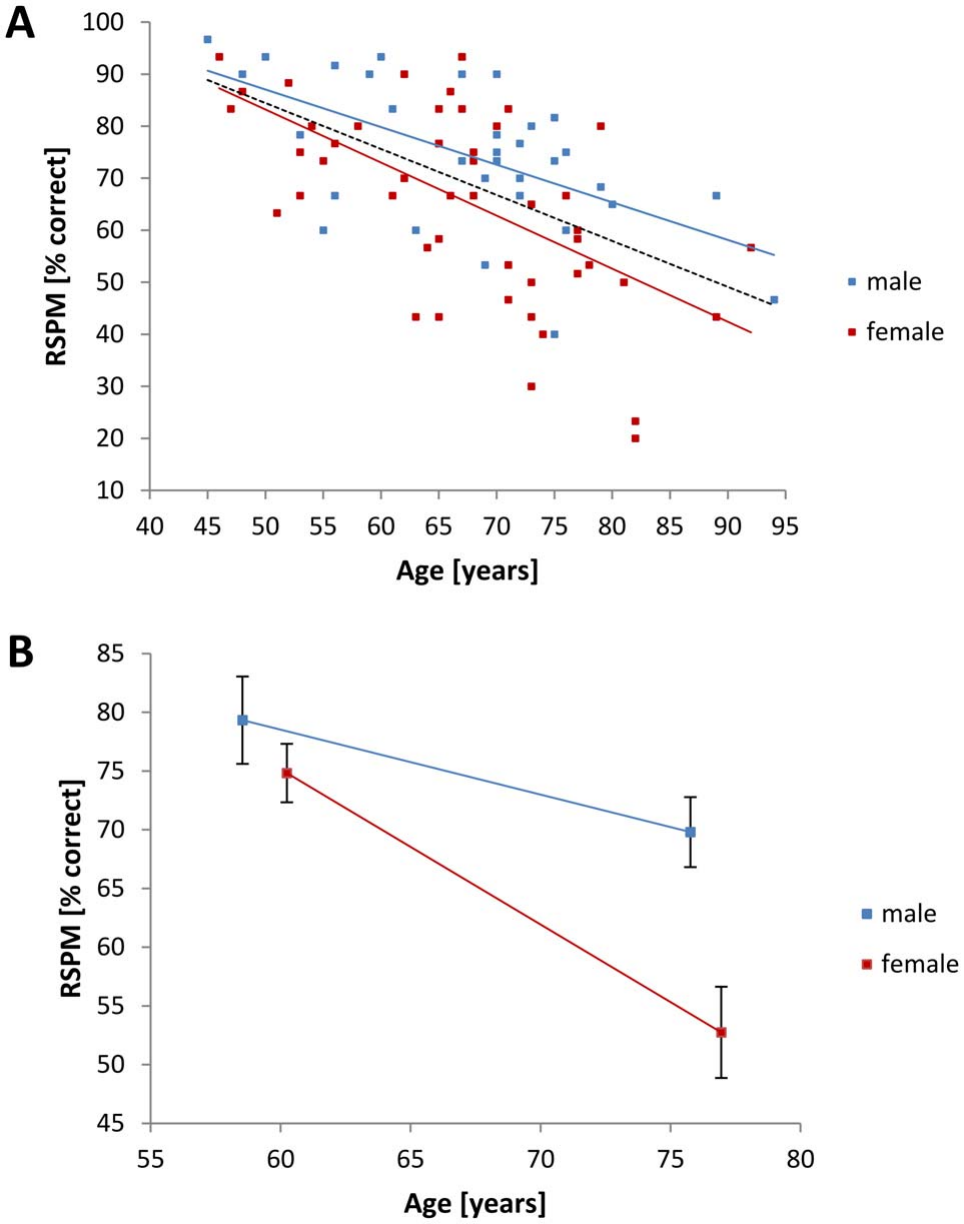
Development of cognitive performance in later life. **A.** Individual cognitive performance scores assessed with the RSPM test (i.e., percent correct answers) are depicted for male (blue squares, N = 32) and female (red squares, N = 49) subjects. Trend lines are shown for male (solid blue), female (solid red), and all subjects (dashed black). Cognitive performance declined with increasing age (Pearson correlation, N = 81, r = −0.550, p≤0.001). **B.** Group data for cognitive performance (RSPM score, percentage correct) for younger adults (left squares; female: N = 29, 60.24±7.07 years; male: N = 15, 58.53±7.62 years) and older adults (right squares; female: N = 20, 76.95±5.94 years; male: N = 17, 75.76±6.71 years). There was no significant interaction of the subjects' age and gender with their cognitive performance (AGE*GENDER: F_(1,31)_ = 17.535; p≤0.001). Black bars indicate SEM.

The subjects' cognitive performance was rated based on the results of the Raven Standard Progressive Matrices (RSPM) test. The percentage of correct answers declined with increasing age (Pearson correlation, N = 81, r = −0.550, p≤0.001) for both male (Pearson correlation, N = 32, r = −0.580, p≤0.001) and female subjects (Pearson correlation, N = 49, r = −0.582, p≤0.001) (**fig. 3A**). The investigation of the subjects' performance revealed a significant interaction for age and gender (AGE*GENDER: F_(1,33)_ = 22.307; p≤0.001) indicating a stronger age-related decline of cognitive performance in female subjects (**fig. 3B**)

### Correlation of haptic, tactile, and cognitive performance

**Figure 4:**
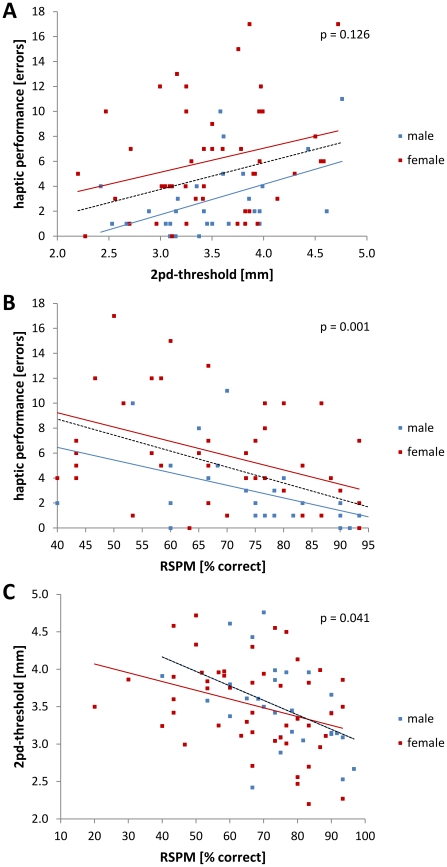
Correlation of haptic, tactile, and cognitive performance. Second order partial correlations controlling for the age of all subjects were calculated. There was no significant correlation between tactile and haptic performance of the subjects (**A**; N = 74, r = 0.133, p = 0.126), but there was a correlation between haptic and cognitive performance (**B**; N = 74, r = −0.348, p = 0.001) and between tactile acuity and cognitive performance (**C**; N = 76, r = −0.198, p = 0.041).

Second order partial correlations controlling for the age of subjects were used to investigate the relationships between tactile, haptic, and cognitive performance. Regarding all subjects' tactile and haptic performance we found no significant correlation (partial correlation corrected for AGE, N = 74, r = 0.133, p = 0.126). In contrast, this correlation was found to be significant in the male subpopulation (partial correlation corrected for AGE, N = 28, r = 0.342, p = 0.032) but not of female subjects (partial correlation corrected for AGE, N = 43, r = 0.063, p = 0.341) (**fig. 4A**). Investigation of the subjects' cognitive and haptic performance revealed a significant correlation between these 2 variables (partial correlation corrected for AGE, N = 74, r = −0.348, p = 0.001). This correlation was also found to be significant in subset of male subjects (partial correlation corrected for AGE, N = 28, r = −0.335, p = 0.036), but not among the female subjects (partial correlation corrected for AGE, N = 43, r = −0.210, p = 0.083) (**fig. 4B**). Finally, we found a significant correlation between the subjects' cognitive performance and tactile acuity as assessed by the two-point discrimination test (partial correlation corrected for AGE, N = 76, r = −0.198, p = 0.041), which was not found in the subset of either the male subpopulation (partial correlation for AGE, N = 28, r = −0.302, p = 0.052) or the female subpopulation (partial correlation for AGE, N = 45, r = −0.153, p = 0.153) (**fig. 4C**).

Comparing the correlation coefficients of haptic and tactile data (N = 74, r = 0.133; **fig. 4A**) with haptic and cognitive data (N = 74, r = −0.348, **Fig. 4B**) we found a significant difference (Fisher-Z-transformation, p = 0.003) indicating a stronger relationship between haptic performance and cognition as compared to haptic performance and tactile acuity. The comparison of the correlation coefficients of haptic and cognitive data (N = 74, r = −0.348, **fig. 4B**) with tactile and cognitive data (N = 76, r = −0.198, **fig. 4C**) revealed no significant differences (Fisher-Z-transformation, p = 0.330).

## Discussion

It has been known for some time that human haptic performance decreases as a function of age [16], but less is known about the neural mechanisms underlying these changes. In a study about haptic performance of adults under conditions were finger movements were restricted, or subjects had to wear gloves, the speed of object identification was more affected than accuracy of identification [46]. In contrast, we have shown recently that the aging process affects both, the speed and the accuracy of haptic identification of unfamiliar objects [44], [43] (cf. fig 4a in [44]). In the present study, we investigated age-related changes in haptic performance by combining a haptic task with tests of tactile acuity and of cognitive performance, as the latter abilities are crucial prerequisites for haptic object exploration. Confirming previous findings [45], [44], [43], we found a significant age-related decline in both tactile and haptic performance. Furthermore, the RSPM test confirmed common knowledge of an age-related decline in cognitive abilities in old age [18], [19]. Correlational analyses revealed a strong relationship between individual cognitive and haptic performance, but only relatively minor relationships between individual tactile and haptic performance, as in the present study only data from male subjects reached significance criteria. In female subjects, who generally showed a stronger decline in haptic and cognitive performance, no relationship between tactile acuity and haptic performance was found. Our results indicate that the well-documented loss of tactile acuity in old age [45], [67], [68], [43], [44], [37], [69], [38]–[41] might not be the primary cause of the age-related decline in haptic performance in later life. Instead, intellectual functioning seems to be more predictive than the sensory measure. This finding has strong implications for the view that aging is associated with greater correlations between intellectual status and sensorimotor performance [54], [55]. Generally, the influences of healthy aging on haptic performance are hard to reveal as they may vary depending on the experimental set-up that is used (pure haptic or cross-modal testing), the objects used (common or unfamiliar, cubic or natural shape), and other boundary conditions (same/different or matching task; time constraints).

### Tactile acuity as a prerequisite for haptics

From previous work in older adults, we know that human tactile, haptic, and fine-motor performance decreases as a function of age, but can be restored to some extent by physical intervention programs [70], [71], or by focused peripheral stimulation paradigms [44], [43], [45], [72]. Under such conditions, the stimulation-based improvement of tactile acuity was shown to support exploratory procedures in haptic object exploration and object manipulation in fine-motor tests [43], [44]. From experiments in healthy adults [73] older adults [38] and patients suffering from impaired tactile perception following central [74] or peripheral neurological disorders [75], it is known that tactile acuity is indispensable for object manipulation, as well as for object recognition. Dellon and Kallman [75] investigated functional sensation in the hands of patients with functional limitations of the median nerve, and found that the moving two-point discrimination test best correlated with the patients' ability to identify objects using their fingertips. Furthermore, the time required for object recognition correlated best with the static two-point discrimination test [75]. From our own experiments, we know that the two-point discrimination paradigm provides very accurate and reliable data for performance on stimuli discrimination tasks [45], [67], [76]–[78], [44], [37], [43]. In a recent study, Legge and coworkers investigated tactile acuity over the entire human lifespan in sighted and blind individuals by means of newly designed tactile-acuity charts that require active exploration [79]. The authors demonstrated good quantitative agreement between their data acquired in sighted subjects and the data of other studies, thereby providing a degree of validity for their measurement technique. Further experiments are required to investigate the relationship between data acquired by common measures of tactile acuity (i.e., the grating orientation test or the two-point discrimination test), haptic object exploration performance and the abovementioned new tactile acuity charts.

### Gender-specific differences in haptic performance

While our results revealed a poor haptic performance of female subjects as compared to male subjects, other studies reported that woman at all age consistently outperformed men in fine dexterity tasks [80], [81]. Kleinman and colleagues, who performed an early investigation into haptic exploration performance in young, middle-aged, and elderly adults, reasoned that the documented loss of performance in old age is due to less logical, systematic, and detailed exploratory procedures that were applied by elderly subjects [16]. As such, older subjects, when asked to identify geometric objects in a purely haptic experiment, seemed to use inappropriate exploratory procedures, which harmed their object recognition. This finding was supported by self-reports from a number of female subjects in the present study, who indicated that they were hardly able to match the haptic impression with the respective visual perception, because they did not know which exploratory procedure to apply. Although all subjects were informed about object-related structural cues (e.g., nubby upper side and plain lower side) and the constructional properties of the used objects (that were additionally highlighted in terms of color), female subjects often failed to identify the explored objects. Male subjects, even those who showed relatively poor haptic performance, did not indicate any problems with handling of the objects.

Our findings are in line with neuropsychological research on visuospatial tasks, particularly those that require mental rotation of objects, as extremely consistent gender differences have been found in these studies [82], [83]. Mental rotation involves the active manipulation of objects in the mind, a process that is based on visuo-spatial memory functions [84], i.e., shape perception, spatial reasoning, and problem solving [85]. Several studies have found males to perform better than females in mental rotation tasks [86], [87] although it remains unclear which specific biological or environmental factors cause women's poorer performance on such tasks [83]. The demonstrated significant correlation between haptic performance and general intelligence, as assessed by the RSPM test, is also supported by a positive correlation between visuospatial and mathematical abilities with respect to gender differences [88], [89]. Besides evolutionary and hormonal mechanisms contributing to the reported robust gender-specific differences, one has also to consider the effects of gender role socialization on spatial ability [90].

From our experiment, we conclude that comprehension of the geometric structure of an object is the first requirement for haptic exploration. This process seems to be a demanding intellectual task in old age, with subjects with high RSPM-scores faring the best. Specifically, it is necessary to comprehend the global geometric structure of the visually presented sample objects, as well as the structure of the haptically explored object. Mental rotation skills are required throughout the process, as the subjects have to align the explored object in their hand relative to the visually presented objects. Once the alignment is completed, the tactile acuity of the fingertips, as assessed by the two-point discrimination test, seems to be the secondary requirement for successful execution of the task. The subjects have to check for characteristic details of the explored object in their hand and assign it to one of the presented objects. This assumption is supported by data from the correlation analyses, where only the data from male subjects, who performed better on average, showed a significant relationship between tactile acuity and haptic performance. In female subjects, who seemed to have more problems with the comprehension of global object structure and the alignment of object orientation, tactile acuity seems to play a subordinate role. It is an interesting remaining question in how far the observed gender differences in haptic identification of unfamiliar objects might to some extend be caused by the fact that we used arbitrary instead of familiar objects. It is conceivable that male subjects, who are typically more frequently exposed to manual tasks associated with manipulating tools or office objects either during work or free time, which might have translated into an advantage performing the haptic task. In fact, it was shown recently that object familiarity modulates the relationship between visual object imagery and haptic shape perception [91]. Accordingly, further studies are needed using familiar objects, although this poses problems because most familiar objects are heavily overlearned.

Our findings are partially in line with the findings of Norman and colleagues, who compared pure haptic, pure visual and cross-modal object recognition in younger and older adults [17]. In a same/different shape discrimination task, they found a strong age-effect for the cross-modal haptic performance, which was independent of the subjects' individual tactile acuity, again assessed with a two-point discrimination test [17]. These observations were reproduced in a more recent work of the authors investigating age-related changes in the haptic perception of three-dimensional surface shape [69]. The absence of correlation between tactile acuity and haptic performance in these studies might be attributable in part to the objects used, that were larger than the objects used in our current experiment. Furthermore, it is possible that proprioceptive functions contribute to the haptic exploration of larger objects (e.g. bell peppers [17]) or single object features (e.g. surface shape [69]). Using a proprioceptive hand function test recently developed by our group (to be published), we found that proprioceptive functions of the human hand are subject to only minimal age-related changes as compared to the dramatic changes in tactile acuity [45], [67], [68], [43], [44], [37]–[41]. The unfamiliar, cubic objects used in our study require tactile acuity to perceive the different surface textures of the upper and lower sides (see methods section), and to align the object in the hand accordingly for exploration. Furthermore, some constructional differences between the 5 classes of objects used are based on tiny details in the lower centimeter-range, which require at least basic tactile acuity.

### Sensory-cognitive link in later life

The results reported herein support recent findings of a sensory-cognitive link found in the auditory, visual, and tactile domains in healthy aging adults [66], [92], [53]. Furthermore, our findings are in line with studies that investigated task complexity and the sensory-cognitive link in old age, as it is commonly found that more cognitively demanding tasks correlate more highly with measures of intelligence (“g”), than do tasks measuring simple sensory detection thresholds [93]–[95]. In the above-mentioned domains, basic measures of absolute sensory thresholds, such as pressure threshold sensitivity, hardly rely on cognitive resources. However, more cognitively demanding processes, such as the current haptic task, which involves processes of comparison, judgment, and mental rotation, show higher correlations with intelligence (“g”) [92].

### Conclusion

We have demonstrated that the age-related decline of cross-modal haptic performance, which occurs in late adulthood, is primarily related to cognitive functioning, rather than to tactile acuity. The results presented here support the view that the haptic object exploration process is structured in 2 phases: a first phase where a global understanding of the explored object is built up (“general exploratory procedure,” [5]) and a second phase where specific object details are explored (“specific exploratory procedure,” [5]). Tactile acuity seems to be a predictor of haptic performance, but only if the cognitively demanding first phase is completed successfully.

## Materials and Methods

### Subjects

We tested 81 right handed volunteers aged 45 to 94 years (32 males, mean age 67.69±11.22 years; 49 females, mean age 67.06±10.58 years; t-test: p = 0.800). In all subjects, the educational level (number of school years and training) was balanced (males 12.08±2.12 years; females 11.20±2.52; t-test: p = 0.109). All subjects were neurologically healthy, as assessed by a neurologist. Individuals with polyneuropathy, peripheral nerve lesions, carpal tunnel syndrome, or other neurological disorders were excluded from the study. Eligibility criteria were lucidity, independence in activities of daily living, absence of motor handicaps such as functional impairments due to arthritis, or other causes of joint immobility. Furthermore, medication with central nervous effects in subjects' present or recent reported history (past 5 years) was a criterion for exclusion. Tactile sensitivity of the subjects' hands was checked prior to the experiments as a check for peripheral neuropathies [44]. Additionally, basic cognitive abilities were assessed using the “Mini Mental State Examination” [96]. Subjects with a score lower than 28 points were excluded. This study was approved by the Ethics Committee of the Ruhr-University of Bochum, and all subjects provided written informed consent before participating.

### Cross-modal haptic object recognition test

**Figure 5:**
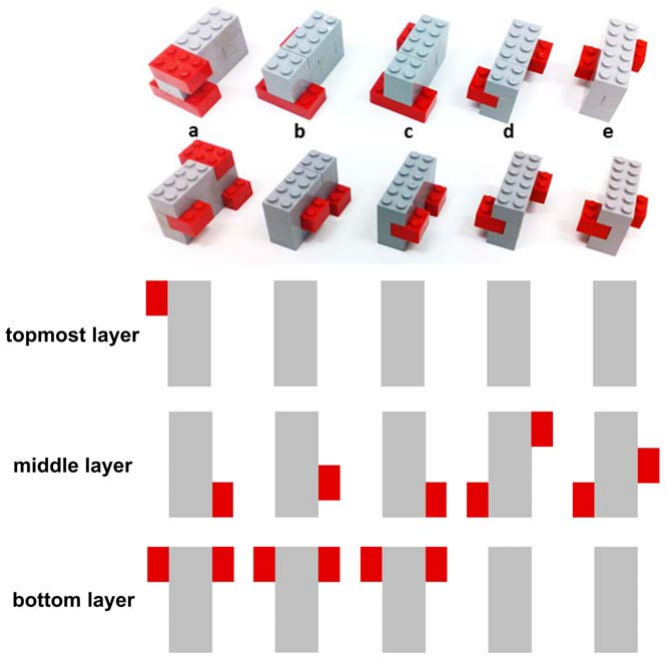
Objects used for the haptic object recognition test. Five groups of unfamiliar objects made from LEGO™ bricks were used for the haptic object recognition test (upper row of photo: view from the backside; lower row of photo: view from the front). In each group, the objects consisted of a cuboid (1.5 * 2.7 * 4.7 cm) with a specific number and position of rectangular structures (each 1.6 * 0.8 * 1 cm (4 in group a; 3 in group b and c, 2 in group d and e)) on the sides (marked in red, c.f. schematic drawing). A total of 17 objects (3 * a, 5 * b, 4 * c, 3 * d, 2 * e) were used for the test.

The custom-made test consisted of 5 different sets of unfamiliar objects made from LEGO™ bricks [44], [43]. The use of unfamiliar, instead of common objects, prevents the influence of prior knowledge of structural information, and creates a comparable initial test-situation for all subjects. Each object was constructed as a cuboid (1.5*2.7*4.7 cm) with an individual number and position of rectangular structures on the sides. These constructional differences were highlighted in terms of color. All objects had a smooth surface structure on the sides, a plain bottom side, and a nubby upper side that could be used as tactile cue for orientation during haptic exploration (**fig. 5**). To prevent the objects from falling apart, all components were glued together. One sample of each set was placed on a desk in front of the subject. The viewing conditions were full-cue, and the objects were binocularly viewed by observers under ample lighting [17]. In a familiarization phase, individual haptic and visual exploration of the objects was allowed. Afterwards, a total of 17 objects, hidden in a small fabric sac (20 * 30 cm), were explored by haptic perception only. For this aim, participants were asked to hold the sac with their left hand underneath the desk, while explorative movements were exclusively performed with the right hand. Each object had to be allocated to one of the visible samples on the desk, by removing it from the sac and placing it in a container behind the specific sample. No visual verification was permitted. The subject was instructed to perform as quickly and as accurately as possible (time limit of 4 minutes per session; remaining objects were considered to be errors). After a familiarization training (3 consecutive sessions), all subjects indicated good comprehension of the test. The estimation of individual performance was done by counting the number of errors occurring in the fourth test session.

### Two-point discrimination test

**Figure 6:**
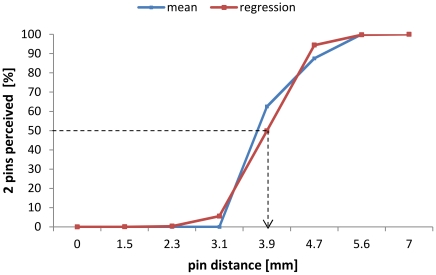
Psychometric function for two-point discrimination performance. Example of the typical two-point discrimination performance of an older adult. Correct responses in percentages (blue squares) are plotted as a function of needle distances. Based on these results, a logistic regression is calculated (red squares). The 50% level of correct responses (dashed line) determines the individual 2-point discrimination threshold (arrow).

Spatial two-point discrimination thresholds were assessed on the tips of all fingers of the right hand using the method of constant stimuli as described previously [44], [45], [97], [77], [76], [78]. We tested 7 pairs of brass needles; in addition, zero distance was tested with a single needle. To overcome problems in the use of two-point measurements associated with hand-held probes, we used a specifically designed apparatus that secures a standardized form of testing (see figures in [45], [67]). The apparatus allowed rapid switching between pairs of needles featuring different separations or 1 single needle (control condition). All tactile stimuli were applied to a fixed position on the skin of the fingertips for approximately 1 s. To account for the age-related decline in tactile acuity [41], [38], [44], [45], [67], we used different settings of the two-point discrimination set-up for subjects below 60 years of age (1.0, 1.4, 1.8, 2.2, 2.6, 3.2, and 4.0) and subjects aged 60 years and older (1.5, 2.3, 3.1, 3.9, 4.7, 5.6, and 7.0 mm). The diameter of the needles was 0.7 mm, and the diameter of the blunt endings was 200 µm. Application force was approximately 150 to 200 mN. Fixation of the tested fingers prevented explorative finger movements. All 8 test conditions were presented 8 times in a randomized order, resulting in a total of 64 tests per session. The subjects, who were not informed of the ratio of needle-pairs to single needles (i.e., 7∶1), had to decide immediately whether they had the sensation of 1 or 2 needles. They were instructed to classify the percept of a single needle or doubtful stimuli as “1” but the distinct percept of 2 stimuli as “2.” The summed responses were plotted against the needle-distances, resulting in a psychometric function, which was fitted with a binary logistic regression (SPSS; SPSS Inc., USA). Threshold was taken from the fit where 50% correct responses were reached (fig. 6). All subjects had to attend 2 training sessions to get used to the testing procedure before the assessment was started in the third session. All subjects who participated in the present study knew the two-point discrimination test from previous studies.

### Raven Standard Progressive Matrices test

The Raven Standard Progressive Matrices (RSPM) test is among the most widely used and researched non-verbal tests of intelligence [98]. Compared to other tests, RSPM scores are recognized as reliable estimates of general intelligence (Spearman's *g* factor) [99]. We applied the paper-and-pencil version of the RSPM to the subjects. The 5 sets (A, B, C, D, and E) of tasks are arranged according to the principles of increasing complexity [100]. In each task, a specific pattern or a number of geometrical structures are presented, with 1 part of the pattern or 1 component of the structures missing. On the basis of 6–8 presented solutions the subject has to decide which one is appropriate to complete the given pattern or set of structures. The RSPM was applied in a “speed-version,” which measures performance within a time limit of 30 minutes (remaining items were considered to be errors).

### Statistical analyses

We investigated age-related changes in haptic, tactile, and cognitive performance using correlational analyses (one-tailed Pearson correlations and second order partial correlations, controlling for factor AGE) and repeated measures ANOVA for factors AGE and GENDER (2 by 2 factorial design). ANOVAs were calculated based on subsamples of the population. We allocated subjects aged 45 to 59 years to the group of “younger adults” (younger female adults “*yf*”: N = 29, 60.24±7.07 years; older female adults “*of*”: N = 20, 76.95±5.94 years) and subjects aged 60 to 94 years to the group of “older adults” (younger male adults “*ym*”: N = 15, 58.53 years; older male “*om*”: N = 17, 75.76±6.71 years).
